# *In vivo* mapping of a dynamic ribonucleoprotein granule interactome in early *Drosophila* embryos

**DOI:** 10.1002/2211-5463.12144

**Published:** 2016-10-25

**Authors:** Jimiao Zheng, Ming Gao, Nhan Huynh, Samuel J. Tindell, Hieu D. L. Vo, W. Hayes McDonald, Alexey L. Arkov

**Affiliations:** ^1^ Department of Biological SciencesMurray State UniversityUSA; ^2^ Biology DepartmentIndiana University NorthwestGaryINUSA; ^3^ Department of BiochemistryMass Spectrometry Research CenterVanderbilt University School of MedicineNashvilleTNUSA; ^4^ Present address: University of AlbertaEdmontonABCanada

**Keywords:** *Drosophila*, germ cells, germ granules, *in vivo* interactome mapping, organelle, Piwi, Tudor

## Abstract

Macromolecular complexes and organelles play crucial roles within cells, but their native architectures are often unknown. Here, we use an evolutionarily conserved germline organelle, the germ granule, as a paradigm. In *Drosophila* embryos, we map one of its interactomes using a novel *in vivo* crosslinking approach that employs two interacting granule proteins and determines their common neighbor molecules. We identified an *in vivo* granule assembly of Tudor, Aubergine, motor and metabolic proteins, and RNA helicases, and provide evidence for direct interactions within this assembly using purified components. Our study indicates that germ granules contain efficient biochemical reactors involved in post‐transcriptional gene regulation.

**Abbreviations**
Aub: Aubergine
eIF4A: eukaryotic translation initiation factor 4A
GAPDH: glyceraldehyde‐3‐phosphate dehydrogenase
Me31B: maternal expression at 31B
piRNA: Piwi‐interacting RNA
PyK: Pyruvate kinase
Tud: Tudor

Cellular life is dependent on the assembly of large macromolecular organelles and complexes which often localize to specific sites inside the cell. In many organisms and cell types, these organelles are frequently dynamic RNP granules which change their architecture during development 1, 2. For example, germ granules are found in germ cells across animal phyla and their identified components play important roles in germline development which ensures the production of gametes and the next generation 1, 3, 4, 5, 6, 7, 8.

Although germ granules were described more than 100 years ago, they have been very challenging to study due to their large size and highly dynamic and complex structure 9, 10, 11. Accordingly, detailed biochemical analysis of the granule assembly mechanisms and systematic mapping of the individual granule components have not been performed. In this work, we have focused on germ granules in early 0–1‐h‐old *Drosophila* embryos referred to as polar granules at this developmental stage. Polar granules are assembled in the egg's posterior cytoplasm known as germ plasm (Fig. 1A,B) which is necessary and sufficient to induce the formation of primordial germ cells at the embryo posterior at ~ 1 h 30 min of embryonic development 12.

**Figure 1:**
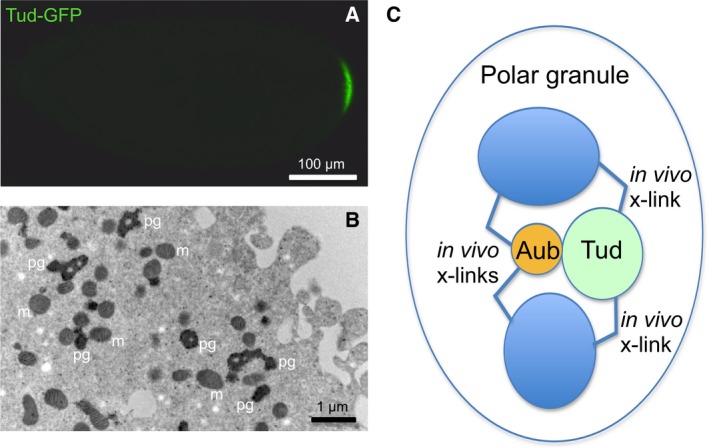
(A) Live imaging of full‐length functional HA‐GFP‐HA‐tagged Tudor localization to germ plasm at the posterior pole of preblastoderm embryo. Anterior is to the left and dorsal is up. (B) Immuno‐EM labeling of polar granules in germ plasm of preblastoderm embryo with anti‐Vasa antibody. pg, polar granules; m, mitochondria. (C) Diagram illustrating *in vivo* crosslinking approach using two interacting polar granule components, Aubergine (Aub) and Tudor (Tud).

Here, we develop an *in vivo* approach to map and position the granule components in living embryos. This approach is based on fast *in vivo* crosslinking of two differently tagged directly interacting granule proteins and their common neighbors within the granules using a low concentration of formaldehyde followed by high‐level purification of the crosslinked complexes and mass spectrometry analysis. Therefore, these two known interacting granule proteins serve as a reliable granule map reference point. Subsequently, the assembly of identified granule components is confirmed with their localization to the granules using immunohistochemistry and *in vitro* reconstitution assays with purified recombinant proteins. In this study, we use the scaffold protein Tudor (Tud) and its interacting partner Piwi protein Aubergine (Aub) as the polar granule reference point (Fig. 1C). Both Tud and Aub are polar granule components essential for germ cell formation during early embryogenesis 13, 14, 15, 16, 17, 18. Furthermore, *tud* and *aub* mutants lack polar granules in the germ plasm 15, 19. Tud protein contains 11 protein–protein interaction modules referred to as Tud domains, and in Tud–Aub complex, Tud domains recognize symmetrically dimethylated arginines (sDMAs) of Aub 20, 21, 22. Also, Aub is associated with small Piwi‐interacting RNA (piRNA) and Aub‐piRNA complex plays a crucial role in the silencing of transposable elements in the germline 23, 24 and RNA localization to the germ plasm 25, 26, 27. In this study, we have mapped motor proteins dynein and kinesin, RNA helicases Me31B and eIF4A and also found unusually high abundance of glycolytic pathway components positioned near Aub–Tud structure within the granules. In addition, we found that RNA helicase eIF4A interacts with both Aub and Tud in *in vitro* binding experiments using purified components. Our data suggest that efficient biochemical reactors are assembled within germ granules to function in post‐transcriptional regulation of gene expression. Furthermore, our study paves the way for *in vivo* mapping and detailed analysis of different cellular granules and organelles.

## Materials and methods

### *Drosophila* lines

Transgenic lines expressing functional full‐length HA‐GFP‐HA‐tagged Tud from *nanos* promoter were generated as described for HA‐tagged Tud‐expressing transgenic lines 13 except GFP and two HA tags that flank GFP insertion were added at the N terminus of Tud. For crosslinking experiments, functional full‐length HA‐Tud 13 and GFP‐Aub 16 were used.

### Crosslinking and purification of Tudor and Aubergine crosslinked complexes

*In vivo* crosslinking and purification of crosslinked complexes were performed as described 28. In particular, 0–1 h embryos expressing HA‐Tud, GFP‐Aub, or HA‐tagged GFP (negative control) were crosslinked with 0.2% formaldehyde, immunoprecipitated with anti‐HA (MBL), or anti‐GFP agarose beads (MBL) in the buffer containing 0.5 m urea, 0.01% SDS and 2% Triton X‐100, rigorously washed in the buffer containing 1 m NaCl and 1% IGEPAL CA‐630 and eluted from the beads with either HA peptide (anti‐HA beads) or SDS/PAGE sample buffer (anti‐GFP beads). HA‐tagged GFP was not crosslinked to any proteins under these conditions 28 and it was used as a negative control for both HA‐Tud and GFP‐Aub crosslinked complexes. Protein complexes were further purified using SDS/PAGE with 3–7% (Tud complexes) or 3–15% (Aub complexes) polyacrylamide step gels. We described step gel system 28 which allows purification and separation of crosslinked complexes, trapped at the low–high polyacrylamide border, from uncrosslinked Tud or Aub proteins. Subsequently, components of purified complexes were subjected to in‐gel trypsin digestion and the resulting peptides were identified by LC‐coupled tandem mass spectrometry followed by database searching against the *Drosophila* database (Mass Spectrometry Research Center at Vanderbilt University Medical Center).

In addition to formaldehyde *in vivo* crosslinking, GFP‐Aub‐expressing embryo lysates were independently crosslinked with an *in vitro* crosslinker, BM(PEG)_3_, [1,11‐bismaleimidotriethylene glycol] using 300 μg·mL^−1^ of the crosslinker as we described for HA‐Tud *in vitro* crosslinking 18, 29. GFP‐Aub crosslinked complexes were immunoprecipitated in PBS containing 0.5 m urea, 0.01% SDS, 2% Triton X‐100, 10% glycerol, 1 mm PMSF and protease inhibitor cocktail (Roche, Indianapolis, IN, USA), washed, eluted, and gel‐purified as described above for *in vivo* formaldehyde‐crosslinked complexes.

### Immunohistochemistry

These procedures have been described 29, 30. The following antibodies were used for whole‐mount embryo immunostaining: rabbit anti‐Vasa antibody (1 : 2000) 30 and mouse anti‐dynein heavy chain antibody (Dhc) 2C11‐2 (1 : 1000; Developmental Studies Hybridoma Bank, Iowa City, IA, USA).

Preparation of the preblastoderm wild‐type (Oregon‐R) embryos for immuno‐EM has been described 18, 29 and we have used the anti‐dynein intermediate chain monoclonal antibody (MAB1618, EMD Millipore, Billerica, MA, USA, Chemicon Trade Name) to detect dynein intermediate chain in *Drosophila* 13.

### Image analysis and statistics

Dhc distribution in preblastoderm and blastoderm early embryos was analyzed with imagej software (https://imagej.nih.gov/ij/). Specifically, for a given embryo, fluorescence intensity from an optical section area of 216–219 pixels corresponding to a germline locus (germ plasm, pole buds or germ cells) and from the same area in the somatic cortex of the same embryo was measured individually for each pixel. Unpaired two‐tailed *t*‐test analyses were performed to determine whether the differences in pixel intensities from germline loci and soma are statistically significant. Optical sections from 13 embryos were analyzed.

### Protein expression and purification

Recombinant Tud, Aub, eIF4A, and eGFP as a control for *in vitro* binding experiments were expressed in Sf9 (Tud and Aub) or S2 cells (eIF4A and eGFP) as follows. Myc‐tagged eIF4A construct was described and kindly provided by Run Shen and Ting Xie 31. eGFP was cloned into Gateway system pAFMW vector (developed by T. Murphy laboratory and available from Drosophila Genomics Resource Center) and this construct had N‐terminal 3xFLAG‐6xMyc tag. Tud had N‐terminal His‐ and HA‐tags and we described its expression in Sf9 cells and purification with Ni‐nitrilotriacetic acid purification system 29. For Aub expression, *aub* cDNA was cloned into pFastBac/NT‐TOPO vector (Thermo Fisher Scientific, Waltham, MA, USA) and subsequently an expression bacmid was generated for Sf9 cell transfection according to manufacturer's instructions. Aub expression construct had N‐terminal His‐, Myc‐, and 3xFLAG‐tags.

Typical protein purification from S2 cells was carried out as follows. eIF4A or eGFP‐expressing cells (~ 50 μL) were lysed in 500 μL lysis buffer (PBS, 0.1 m glycine, 1 m NaCl, 1% IGEPAL CA‐630 (Sigma, St. Louis, MO, USA), 0.1% Tween 20, and protease inhibitor cocktail, Roche) with 15 strokes using a pellet pestle. Cell lysates were centrifuged at 16 000 ***g*** at 4 °C for 30 min and the supernatant was kept for protein purification. Specifically, 20 μL of anti‐c‐Myc beads (MBL) were added to 500 μL of the supernatant and incubated on a rotary shaker at 4 °C for 60 min, followed by washing six times with lysis buffer. The eIF4A or eGFP proteins were eluted from the beads with 20 μL of c‐Myc peptide in PBS (1 mg·mL^−1^) (MBL) twice at 4 °C for 5 min. The purified proteins were stored at 4 °C until use.

For Aub purification, Sf9 cells (~ 1 mL), transfected with *aub* bacmid, were thawed on ice for 30 min. Subsequently, the cells were homogenized in 6 mL lysis buffer (PBS, 0.1 m glycine, 1 m NaCl, 1% IGEPAL CA‐630 (Sigma), 0.1% Tween 20 and protease inhibitors, Roche) with 15 strokes using a glass tissue homogenizer. Cell lysate was centrifuged at 16 000 ***g*** at 4 °C for 30 min, and the supernatant was kept for further purification as follows. Twenty‐five microliters of anti‐c‐Myc beads were added to every 1 mL of the supernatant and incubated on a rotary shaker at 4 °C for 60 min, followed by washing six times with lysis buffer. Aub was eluted from the beads with 25 μL of c‐Myc peptide as described above for the elution of eIF4A and eGFP.

### *In vitro* binding assays

For Aub‐eIF4A‐binding assay, purified Aub or eGFP control were bound to 12.5 μL of anti‐FLAG tag beads (anti‐DDDDK tag beads, MBL) in lysis buffer (PBS, 0.1 m glycine, 1 m NaCl, 1% IGEPAL CA‐630 and 0.1% Tween 20). The prepared beads were washed once with cold PBS and equilibrated with cold PBS, 0.05% IGEPAL CA‐630. Equal amounts (200 μL) of purified Myc‐tagged eIF4A in PBS, 0.05% IGEPAL CA‐630 were added to the Aub and GFP beads and incubation was carried out at 4 °C for 60 min with gentle mixing every 15 min. Subsequently, the beads were washed three times with cold PBS, 0.05% IGEPAL CA‐630 and proteins were eluted from the beads with SDS/PAGE sample buffer at 95 °C for 5 min. Eluted proteins were detected in a western‐blot experiment using anti‐c‐Myc antibody (Santa Cruz Biotechnology, 1 : 1000).

Tud‐eIF4A‐binding assays were carried out with similar approach and conditions used for Aub‐eIF4A‐binding experiments. However, in this case, Myc‐tagged eIF4A and eGFP control were added to HA‐Tud bound to anti‐HA beads (MBL). In additional experiments, purified HA‐Tud was added to eIF4A or eGFP bound to anti‐c‐Myc beads (MBL). In all these experiments, Tud‐eIF4A‐specific interaction was observed. HA‐Tud was detected in western blot experiments with anti‐HA antibody (Roche, 1 : 2000).

## Results and Discussion

### *In vivo* mapping of germ granules

Our systematic approach to map germ granules is based on using different interacting *bona fide* granule components as a reference point for the identification of their molecular neighbors at a specific developmental stage. In particular, to map molecular neighborhoods in large granule assembly with high degree of confidence, we design a general strategy which interrogates whether the same molecule crosslinked *in vivo* to granule component 1 is also independently crosslinked to granule component 2 that is known to directly interact with component 1 in the granules. Subsequently, localization of the identified molecules to germ granules is verified with immunohistochemistry and their interactions are tested *in vitro* using purified recombinant proteins. In this study we are using interacting germ granule proteins Aub and Tud as the granule's map reference point (Fig. 1).

For *in vivo* crosslinking we use short reaction time (10 min) and a low concentration of formaldehyde (0.2%) which efficiently crosses the cell membrane and has a short crosslinking range of 2.3–2.7 Å 28. These conditions enable fast and specific crosslinking and never result in crosslinking of HA‐tagged GFP expressed in the embryos which is used as a negative control in all our experimental steps including mass spectrometry identification of rigorously washed and gel‐purified crosslinked germ granule complexes (Materials and methods, 28). Also, formaldehyde can quickly crosslink neighboring molecules under physiological conditions 32, thereby effectively ‘freezing’ the granule complexes at their native configuration at a given developmental stage.

**Figure 2:**
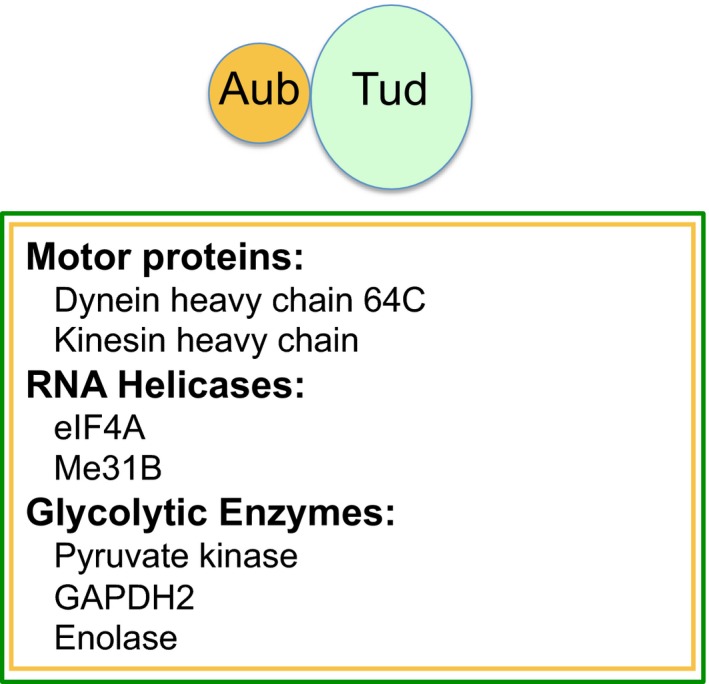
Motor proteins, RNA helicases and glycolytic enzymes associate with Aubergine and Tudor in living embryos. Proteins identified in both Aub and Tud complexes in 0–1‐h‐old embryos with *in vivo* crosslinking approach are shown. Also, refer to Table 1 for details of the protein identification.

First, we purified crosslinked Aub complex from 0 to 1 h‐old embryos with polar granule‐localized functional GFP‐tagged Aub and identified the components by mass spectrometry. Independently, we also identified proteins crosslinked to functional HA‐tagged Tud under the same conditions. We found that motor protein components dynein heavy chain 64C (Dhc) and kinesin heavy chain, RNA helicases eIF4A and Me31B, and glycolytic enzymes Pyruvate kinase (PyK), glyceraldehyde‐3‐phosphate dehydrogenase 2 (GAPDH2) and Enolase (Eno) were crosslinked to Aub and Tud *in vivo* (Fig. 2 and Table 1).

**Table 1:**
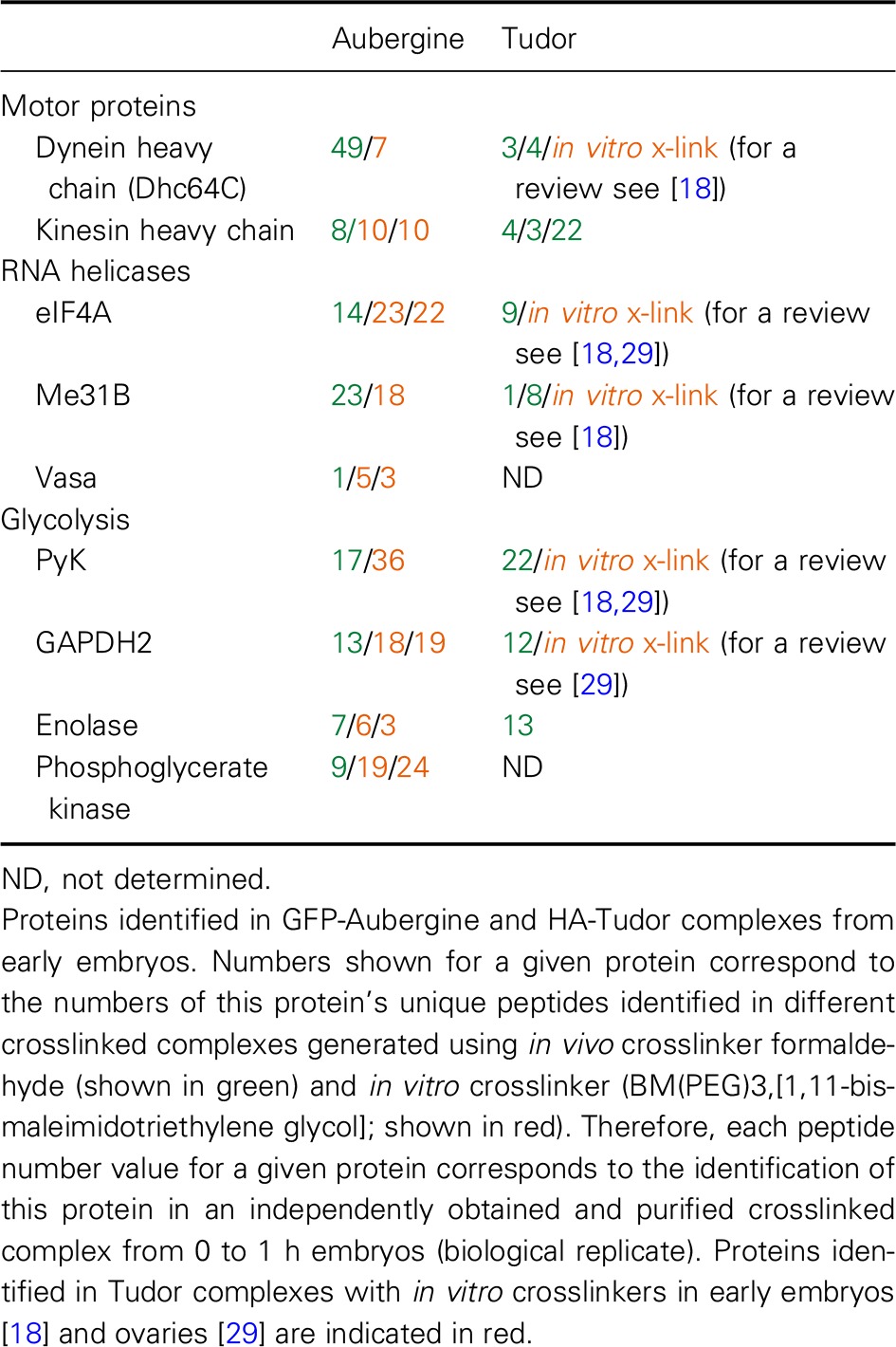
Components of the Aubergine and Tudor complexes identified with crosslinking approaches

Next, we asked whether crosslinkers of different chemistry and crosslinking mechanism would be able to confirm the components of Tud–Aub molecular neighborhood. Indeed, using an *in vitro* crosslinker BM(PEG)_3_,[1,11‐bismaleimidotriethylene glycol] which interacts with sulfhydryl groups of cysteine residues, we showed that all these components are crosslinked to Aub (Table 1). Similarly, the majority of these components were crosslinked to Tud in *in vitro* crosslinking experiments (Table 1).

### Tud–Aub molecular neighborhood

#### Motor proteins

Motor proteins were identified in Tud–Aub complex (Fig. 2 and Table 1). Consistent with our data, important roles of dynein and kinesin in motility of germ plasm at the posterior of *Drosophila* egg were reported and dynein is also crucial for long‐term retention of germ plasm in the egg 33. Later, during germ cell formation in *Drosophila* embryos, dynein‐dependent transport ensures segregation of germ plasm components into forming germ cells 34. However, it has not been clear whether dynein is an integral component of polar granules in *Drosophila* and if it is, where and when it is located in the granules. Here, we provide *in vivo* evidence that dynein motor is a *bona fide* polar granule component and it is positioned close to Tud–Aub in the granules before it is required for the transport of germ plasm during germ cell formation.

**Figure 3:**
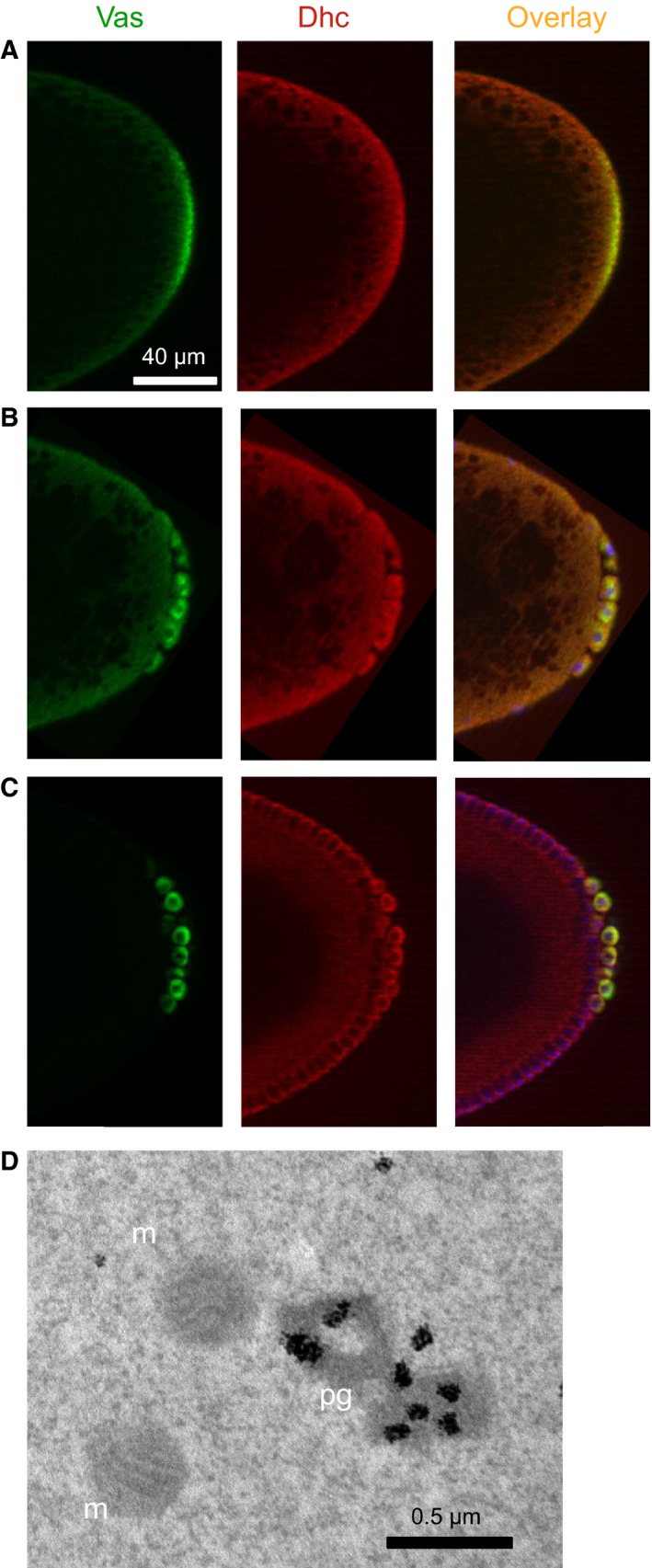
Dynein motor is a *bona fide* polar granule component. (A) Dynein heavy chain (Dhc) colocalizes with polar granule marker Vasa in the germ plasm of early preblastoderm embryos. (B) During initial stages of germ cell formation, posterior nuclei enter germ plasm and are incorporated into pole buds with polar granule material. Dhc is frequently observed to be slightly enriched in pole buds (see Results and Discussion). (C) Dhc continues its small enrichment and colocalization with Vasa in the formed germ cells at the posterior of blastoderm embryos. In (A)–(C), wild‐type embryos were costained with rabbit anti‐Vasa antibody (green channel) and mouse anti‐Dhc antibody (red channel). Overlay images are shown and in (B) and (C) they include DAPI costaining to show the nuclei which have migrated to the cortex of the embryos (blue channel). Posterior is to the right. Forty micrometer scale bar in (A) is the same for (B) and (C). (D) Immuno‐EM image of the preblastoderm embryo germ plasm stained with anti‐Dynein Intermediate chain (Dic) antibody which strongly labels polar granules (pg) demonstrating that Dic is a polar granule component. m, mitochondria; scale bar is 0.5 μm.

First, we determined distribution of Dhc in early embryos and asked whether Dhc may be enriched in germ plasm (Fig. 3A), pole buds (Fig. 3B), and germ cells (Fig. 3C). To this end, we quantified optical sections from 13 preblastoderm and blastoderm embryos after staining with anti‐Dhc antibody as follows. Seven out of nine preblastoderm embryos showed slight but statistically significant enrichment of Dhc in germ plasm and pole buds when compared with Dhc signal in somatic cortex of the same embryos (average enrichment 1.24‐fold ± 0.05 (SEM), *P* < 0.0001). The other two preblastoderm embryos did not show significant enrichment of Dhc in germ plasm and pole buds. In addition, four blastoderm embryos showed enrichment of Dhc in germ cells compared to somatic cortex (average enrichment 1.36‐fold ± 0.05 (SEM), *P* < 0.0001). While this enrichment is small, given that dynein is one of the major motors in both germline and soma, it is expected that Dhc will be detected in substantial amounts in the soma. Dhc slight enrichment bias in germline embryonic loci and colocalization of Dhc and polar granule marker Vasa in these loci are consistent with our identification of Dhc in Tud and Aub complexes in the granules.

Dynein motor is a large multisubunit assembly and if this motor is a polar granule component, besides Dhc, one would expect to detect another dynein subunit in the granules. Therefore, we next asked whether another component of dynein motor, Dynein intermediate chain (Dic) is localized to polar granules. We found that Dic is highly enriched in these granules well before germ cell formation stage as shown with high‐resolution immunoelectron microscopy (EM) technique (Fig. 3D). Gold particles which correspond to the location of Dic on immuno‐EM images were counted and polar granules had 15.9 times more Dic than would be expected from the same germ plasm area if Dic were distributed evenly (285 gold particles from two different EM images counted).

Interestingly, in zebrafish embryos, dynein is also a germ granule component and it is involved in the fragmentation of the granules during germ cell development 35 suggesting that incorporation of dynein in embryonic germ granules has been evolutionarily conserved.

#### RNA helicases and the glycolytic cluster

ATP‐dependent RNA helicases eIF4A and Me31B as well as three glycolytic enzymes have been independently identified in the *in vivo*‐crosslinked Tud and Aub complexes (Fig. 2 and Table 1). These data support our recent study which demonstrates that the glycolytic enzyme PyK is a polar granule component and that several glycolytic enzymes are specifically involved in piRNA biogenesis in the germline and germ cell formation 29. Also, similarly to Aub, PyK contains an sDMA residue and binds directly to Tud domains of Tud protein 29 consistent with its location at Tud–Aub complex shown here. Interestingly, another glycolytic enzyme, Phosphoglycerate kinase (Pgk), was shown to be a polar granule component using immuno‐EM 29, and in this study we identified Pgk in Aub‐crosslinked complexes (Table 1). Therefore, our data point to an unprecedented tight clustering of multiple glycolytic components at Tud–Aub structure in the polar granules. It was recently suggested that another polar granule component ATP‐dependent RNA helicase Vasa (Figs 1B and 3A–C) may use glycolytic ATP during protein synthesis 36. In support of this hypothesis, we detected Vasa in Aub‐crosslinked complexes (Table 1) indicating Vasa proximity to the glycolytic cluster.

**Figure 4:**
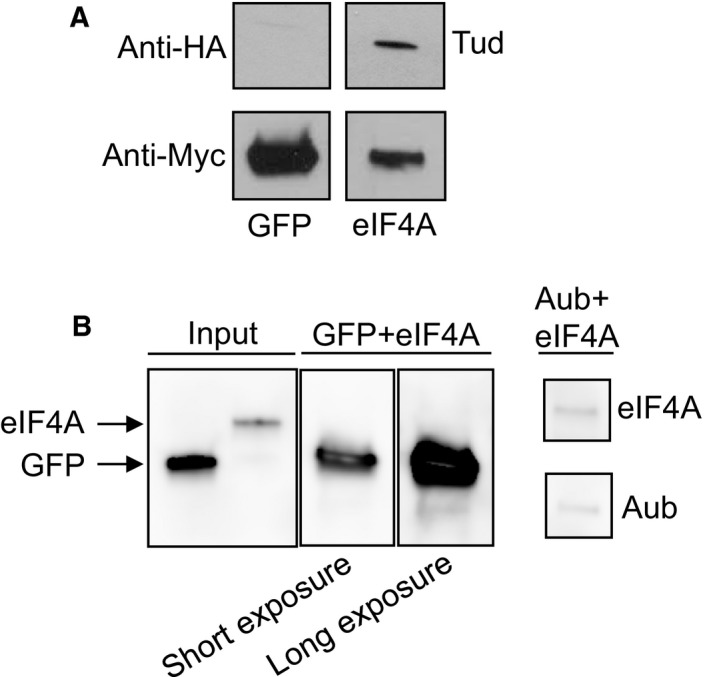
Tudor and Aubergine bind RNA helicase eIF4A *in vitro*. (A) eIF4A binds to Tud. Purified HA‐tagged Tud was added to purified Myc‐eIF4A and Myc‐GFP control bound to anti‐Myc beads. Tud associated with eIF4A (right panel) and failed to bind to GFP (left panel). eIF4A‐Tud binding was observed in three independent *in vitro* experiments. (B) eIF4A binds to Aub. Purified eIF4A was added to purified FLAG‐tagged Aub or to FLAG‐GFP control bound to anti‐FLAG beads. eIF4A associated with Aub (right panel) and failed to associate with GFP control (‘GFP + eIF4A’ panels). All proteins had Myc tag and were detected with anti‐Myc antibody. Control ‘GFP + eIF4A’ lane was overexposed (long exposure), nevertheless, no eIF4A bound to GFP was detected. eIF4A‐Aub binding was detected in two independent *in vitro* experiments.

Supporting our mapping data for the eIF4A and Me31B RNA helicases, both helicases were shown to be polar granule components with immuno‐EM approach and to contribute to germ cell formation presumably by their involvement in RNP remodeling and translational control in the granules 3, 18. However, contrary to PyK‐Tud direct interaction data, biochemical studies to test whether the helicases associate directly with Tud protein scaffold have not been carried out. Therefore, in this study, we focused on testing whether eIF4A interacts directly with Tud using an *in vitro* binding assay and purified components. In particular, we purified recombinant eIF4A and negative control GFP expressed in S2 cells. In addition, recombinant Tud was purified from Sf9 cells using baculovirus expression system 29. We showed that *in vitro* eIF4A specifically binds Tud (Fig. 4A).

Since Aub is involved in different aspects of RNA metabolism, we next asked if RNA helicase eIF4A would interact with Aub, similar to eIF4A binding to Tud. Therefore, we generated recombinant Aub with Sf9‐baculovirus system and tested its interaction with purified eIF4A *in vitro*. Indeed, specific interaction between purified eIF4A and Aub was demonstrated (Fig. 4B). Binding of eIF4A to both Tud and Aub shown here *in vitro* strongly validates our *in vivo* mapping study and provides a rational for future bottom‐up approaches aiming at *in vitro* reconstitution of germ granule structures from purified components to understand their molecular functions.

### Tud–Aub molecular assembly in germ granules

**Figure 5:**
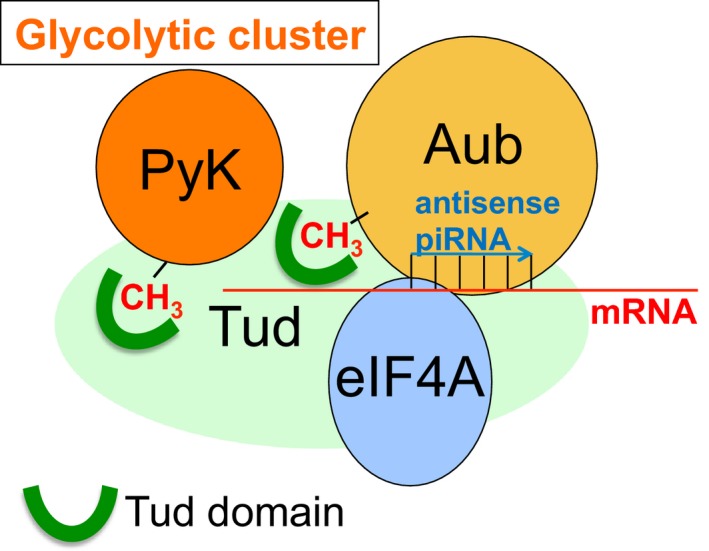
Model of Tud–Aub complex in polar granules. Tud protein is a molecular scaffold which contains 11 protein–protein interaction modules, Tud domains 13, 37. Similar to Aub, glycolytic enzyme PyK contains a symmetrically dimethylated arginine (sDMA) and this enzyme directly interacts with Tud domains 29. RNA helicase eIF4A is recruited to this complex by interacting with Tud and Aub, and may contribute to post‐transcriptional gene regulation by remodeling polar granule RNP (e.g., granule mRNA‐piRNA‐Aub structure) affecting RNA translation in the granules and RNA stability. Since eIF4A requires ATP for its RNA helicase activity, it may benefit from the source of local ATP generated by ATP‐producing enzymes (for example, PyK) of the glycolytic cluster identified at the Tud–Aub structure.

Here, we propose an approach which can generally be used for *in vivo* mapping of multi‐component and dynamic cellular structures at a specific developmental stage. Using this approach, we provide evidence for *in vivo* assembly of a germ granule structure composed of Tud, Aub, motor proteins, RNA helicases, and glycolytic components during the first hour of embryonic development in *Drosophila*. In addition, we demonstrate that RNA helicase eIF4A interacts with both Tud and Aub in this structure. Furthermore, we have recently shown that glycolytic enzyme PyK contains an sDMA and, similarly to Aub, this enzyme binds to Tud domains of Tud protein 29. Figure 5 summarizes our data based on the evidence for direct interactions within Tud–Aub structure and also highlights the unusual abundance of glycolytic enzymes at this structure identified in this study, which we refer to as the glycolytic cluster. The glycolytic cluster is likely to be responsible for the generation of local ATP that can fuel ATP‐dependent components at the structure such as RNA helicases or motor proteins and therefore, may ensure their efficient functioning during RNA localization to the granules, control of RNA translation, and transport of polar granule material to primordial germ cells. However, we cannot rule out other noncanonical and nonenzymatic roles of the glycolytic cluster in the granules. Therefore, the precise involvement of the glycolytic cluster at the Tud–Aub structure in polar granules awaits further investigation.

## Author contributions

ALA conceived and supervised the study; JZ, MG, NH, SJT, HDLV, and ALA designed and performed experiments; WHM carried out mass spectrometry identification of proteins; ALA wrote the manuscript; all authors contributed to manuscript revisions.
